# Mapping International Collaboration and Research Trends in Artificial Intelligence Applications for Liver and Kidney Transplantation

**DOI:** 10.1155/joot/9692976

**Published:** 2026-01-28

**Authors:** Haneen Al-Abdallat, Noor Haj Mohammad, Ayham Asassfeh, Emily Cooper, Ayham Mohammad Hussein, Mohammad AlSarayreh, Mohammad Alzoubi, Badi Rawashdeh

**Affiliations:** ^1^ School of Medicine, the University of Jordan, Amman, Jordan, ju.edu.jo; ^2^ Division of Transplant Surgery, Medical College of Wisconsin, Milwaukee, Wisconsin, USA, mcw.edu; ^3^ Faculty of Medicine, Al- Balqa’ Applied University, Salt, Jordan; ^4^ Department of General Surgery, University of North Carolina, Chapel Hill, North Carolina, USA, unc.edu; ^5^ Department of General Surgery, the University of Jordan, Jordan University Hospital, Amman, Jordan, ju.edu.jo

**Keywords:** artificial intelligence, bibliometric analysis, kidney transplantation, liver transplantation

## Abstract

**Introduction:**

The integration of artificial intelligence (AI) in liver and kidney transplantation (LKT) research has surged in recent years, promising novel approaches to address traditional statistical challenges and enhance result robustness and generalizability. This study aims to explore the extent of international collaboration and the evolution of research trends in AI applications for LKT.

**Methods:**

On August 12, 2025, a systematic search was conducted using the Web of Science database to identify relevant literature. Bibliometric tools, including the “bibliometrix” package in R, VOSviewer, and Microsoft Excel were used. Key indicators such as country contributions, multiple‐country publications, single‐country publications, co‐authorship, and keyword co‐occurrence were examined to assess collaboration patterns and research hotspots. Inclusion criteria involved all published peer‐reviewed articles related to AI in LKT. Editorials, corrections, and irrelevant documents were excluded.

**Results:**

A total of 633 articles published between 1994 and 2025 were included in the analysis. These collectively received 8959 citations. The United States of America emerged as the leading contributor, accounting for 37.12% of the publications, followed by China and South Korea. Notably, international co‐authorship was evident in 30.02% of the publications. Keyword analysis revealed that “survival,” “outcomes,” “risk,” “mortality,” and “prediction” were the most frequent terms, highlighting them as hotspots in transplantation research.

**Conclusion:**

The field of AI in LKT research is characterized by a growing international collaboration, despite the fact that participation is still uneven and concentrated in high‐income countries. In order to advance the field and enhance outcomes across diverse patient populations, it will be crucial to strengthen global data‐sharing and cultivate equity‐focused, culturally adaptable AI models.

## 1. Introduction

Incorporating artificial intelligence (AI) into liver and kidney transplantation (LKT) represents a significant advancement in addressing the complex challenges of end‐stage liver disease (ESLD) and end‐stage renal disease (ESRD) [1]. AI is at the forefront of the next wave of innovations in LKT. It has demonstrated growing value in LKT, such as new predictive models of graft rejection and survival [2–5], complementing advancements such as machine perfusion and improved organ allocation systems. AI’s ability to analyze large datasets, improve donor–recipient matching, and customize treatment plans shows outstanding potential for enhancing decision‐making, improving transplant outcomes, and addressing critical issues such as donor shortages and chronic rejection [1, 6]. For example, machine learning and deep neural networks have been applied to select suitable donors and recipients more efficiently, predict postoperative complications, and tailor management plans to those patients [7–10].

Given the substantial genetic and geographical variations in LKT‐related diseases, international collaboration and data sharing are essential. Such collaboration enables the development of genetically informed AI models that are globally applicable, increasing the accuracy and relevance of AI tools in predicting LKT outcomes. Leveraging the diversity of LKT patients worldwide, international partnerships can drive the creation of tailored, effective, AI‐driven solutions, ultimately improving patient care on a global scale.

This bibliometric study aims to systematically analyze and map international collaborations and research trends in the evolution of AI utilization in LKT. By examining co‐authorship networks, institutional partnerships, and publication trends, this study illuminates the current landscape of global efforts in employing AI for LKT. It also identifies emerging trends and points out the importance of collaborative initiatives in advancing the field. This study addresses a key gap in the literature by quantifying global research dynamics and international collaborations, providing a data‐driven overview of efforts in this domain.

## 2. Methods

### 2.1. Database Selection and Search Strategy

The Web of Science (WoS) database was used for its comprehensive bibliometric coverage, including titles, authors, countries, affiliations, and related data. A systematic search was conducted on August 12, 2025, using advanced queries to identify AI‐related studies in LKT literature. Only original articles were included, yielding 633 relevant documents for analysis (Figure 1). Inclusion criteria involved peer‐reviewed publications related to AI in LKT without language restrictions, while editorials, corrections, and irrelevant documents were excluded.

**Figure FIGURE 1:**
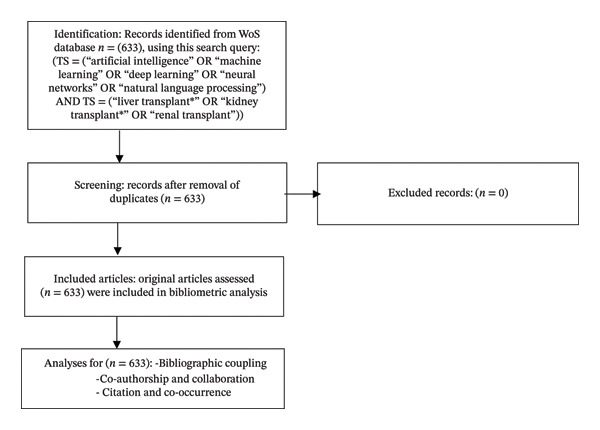
Flow diagram of the literature search, selection, and analysis process.

The final advanced search query was (TS=[“artificial intelligence” OR “machine learning” OR “deep learning” OR “neural networks” OR “natural language processing”] AND TS=[“liver transplant∗” OR “kidney transplant∗” OR “renal transplant”]).

WoS was chosen due to its superior accuracy and quality of citation metrics, extensive coverage of highly indexed journals, reduction of duplicates and irrelevant data, and comprehensive analytical features that enable the generation of bibliometric indicators, including trends in annual publications and citations [11, 12].

This study was exempt from institutional review board approval, as it solely involved bibliometric analysis and did not involve the extraction of patient data.

### 2.2. Bibliometric Analysis

Following the search, data were extracted, and a bibliometric analysis was performed. Descriptive analyses of bibliometric indicators were performed using the “bibliometrix” package in the R programming language (Version 4.2.2), VOSviewer (Version 1.6.20), and Microsoft Excel Office 365. The analysis included country contributions, international collaborations, and frequently co‐occurring keywords. Co‐authorship and bibliographic coupling analyses were conducted to identify major contributors and intercountry connections. Collaboration patterns were assessed using two bibliometric indicators: Multiple‐country publications (MCPs) and single‐country publications (SCPs). SCP refers to articles in which all contributing authors are affiliated with institutions from the same country, reflecting domestic research collaboration. In contrast, MCP represents articles co‐authored by researchers affiliated with institutions from two or more different countries, indicating international collaboration. These indicators were generated using the bibliometrix package in R.

### 2.3. VOSviewer Visualization Interpretation

VOSviewer, a tool for creating bibliometric networks, was used to visualize the data. It represents terms as circles with sizes indicating their weight (occurrences/citations). Connections and co‐citation relationships between terms are shown through lines and proximity, while different colors denote clusters and publication years in overlay visualizations. Total link strength (TLS) is the cumulative weight of all connections an item has with other items in a bibliometric network, reflecting its overall degree of interaction or influence.

## 3. Results

A total of 633 articles published between 1994 and 2025 were included, originating from 307 sources (including journals, books, and other scholarly outlets). All articles were published in English, with the exception of one Spanish and one Chinese publications, which were translated into English. The earliest indexed study in the WoS database documenting AI applications in LKT was published in 1994. The dataset demonstrated an annual growth rate of 16.64%, with an average article age of 3.37 years, and a mean of 14.16 citations per article. Collectively, the articles cited 20,429 references and included 2493 keywords. The average number of co‐authors per article was 9.12, and international collaboration accounted for 30.02% of all authorship activity (Table 1).

**Table TABLE 1:** Summary of articles’ characteristics and types in AI research for LKT.

Description	Results
Timespan	1994:2025
Sources (journals, books, etc.)	307
Annual growth rate %	16.64%
Article average age	3.37
Average citations per article	14.16
References	20,429
Keywords	2493
Number of authors	4458
Single‐authored articles	8
Co‐authors per article	9.12
International co‐authorships %	30.02%

Among the contributing countries, the United States of America led with the highest number of articles (*n* = 173), of which 31.8% were MCPs, followed by China (*n* = 155) with a markedly lower MCP proportion (11%). South Korea produced 36 articles, with only 2.8% involving international collaboration. In contrast, several countries demonstrated high proportions of internationally co‐authored research despite lower publication counts. Notably, Canada (68.8%), Belgium (66.7%), Saudi Arabia (66.7%), France (62.5%), the United Kingdom (62.5%), and the Netherlands (60%) exhibited the highest MCP percentages. Germany, Italy, and Australia also showed moderate levels of international collaboration, with MCP rates of 58.6%, 46.2%, and 50%, respectively. Countries such as Japan and Egypt produced research exclusively through SCP, with MCP values of 0% (Figure 2 and Table 2).

**Figure FIGURE 2:**
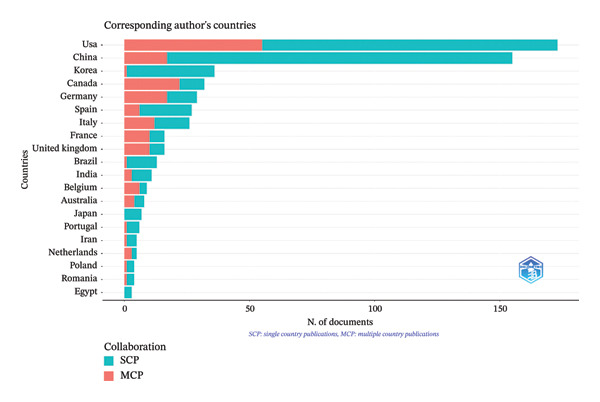
Articles distribution according to country of correspondence and single‐country publications (SCPs) vs. multiple‐country publications (MCPs).

**Table TABLE 2:** Articles distribution according to country of correspondence and single‐country publications (SCPs) versus multiple‐country publications (MCPs).

Country	Articles	SCP	MCP	MCP% from total articles
USA	173	118	55	31.8%
China	155	138	17	11%
South Korea	36	35	1	2.8%
Canada	32	10	22	68.8%
Germany	29	12	17	58.6%
Spain	27	21	6	22.2%
Italy	26	14	12	46.2%
France	16	6	10	62.5%
United Kingdom	16	6	10	62.5%
Brazil	13	12	1	7.7%
India	11	8	3	27.3%
Belgium	9	3	6	66.7%
Australia	8	4	4	50%
Japan	7	7	0	0%
Portugal	6	5	1	16.7%
Iran	5	4	1	20%
Netherlands	5	2	3	60%
Poland	4	3	1	25%
Romania	4	3	1	25%
Egypt	3	3	0	0%
Saudi Arabia	3	1	2	66.7%

In terms of citation, the United States of America produced a total count of 235 publications, accumulating 2469 citations with an average of 14.3 citations per article. The Netherlands recorded the highest average citations per article (58.4 citations), while Indonesia recorded the lowest (1 citation) (Figure 3). Articles originating from the United States of America demonstrated the highest (TLS: 762), while articles from the United Arab Emirates displayed the lowest (TLS: 2). Collaboration analysis revealed that the strongest co‐authorship links were established between the United States of America and Canada, Germany, Italy, Thailand, the United Kingdom, and China, with a frequency of 30, 19, 15, 15, 15, and 13, respectively (Table 3).

**Figure FIGURE 3:**
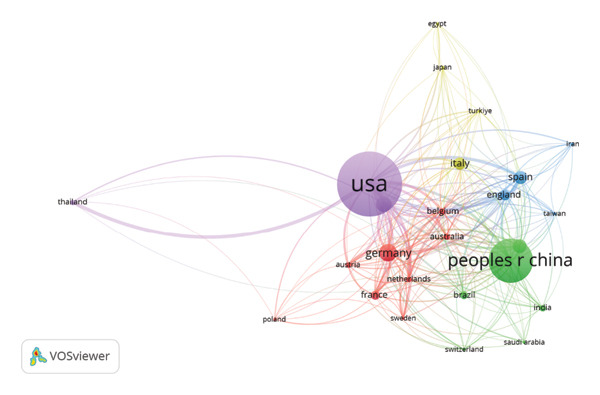
The network visualization of the top contributing countries (according to the number of citations). Countries included in the same cluster are displayed in the same color. Larger circles indicate that the country had more publications. The distance between two circles shows the degree of connection between the two countries.

**Table TABLE 3:** Countries’ distribution according to collaboration frequency among them.

From	To	Frequency
USA	Canada	30
USA	Germany	19
USA	Italy	15
USA	Thailand	15
USA	United Kingdom	15
USA	China	13
Germany	Austria	12
Germany	Belgium	11
USA	Austria	11
Canada	Germany	10
USA	Spain	10
Germany	Netherlands	9
USA	Belgium	9
USA	France	9
USA	Netherlands	9
Canada	Austria	8
Germany	Italy	8
Spain	France	8
USA	Brazil	8

The most frequently used keywords were survival (90), outcomes (65), and risk (64). Those three keywords also had the highest TLS of 392, 284, and 269, respectively. Other keywords used included model, classification, mortality, and prediction (Figure 4a and 4b). Thematic clustering of the keywords revealed five main clusters: the blue cluster focuses on outcome prediction and survival models; the red cluster highlights the pharmacological role in LKT; the green implies risk stratification and outcomes; the yellow underscores AI models in LKT; and the purple cluster denotes oncologic consideration in the context of liver transplantation, particularly hepatocellular carcinoma.

Figure FIGURE 4(a) Network visualization of the top‐occurring keywords and their interconnections grouped in five clusters, where each color represents a cluster of related items. Larger circles indicate that the keyword appears more frequently. The distance between the two circles shows the degree of connection between the two keywords. (b) Overlay presentation of the top‐occurring keywords and their interconnections across the years. The color of a term indicates the average publication year. A color bar is shown in the bottom right corner of the visualization that indicates the publication year range.

**Figure:**
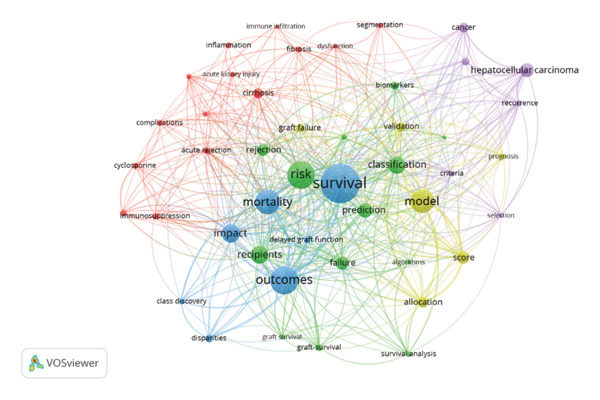
(a)

**Figure:**
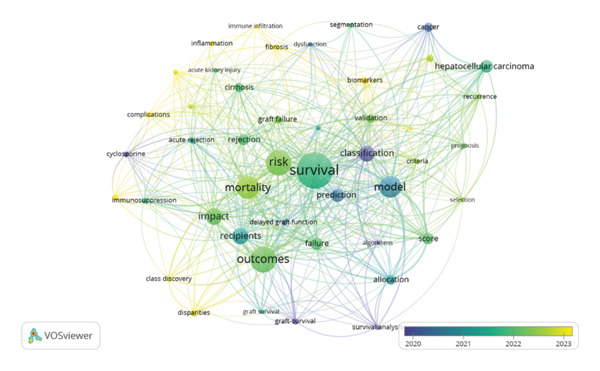
(b)

## 4. Discussion

AI has constantly demonstrated its ability to overcome statistical obstacles, manage large datasets, identify patterns, and generate more reliable results across medical research [13]. The integration of AI in LKT has grown substantially in the last decade, optimizing donor–recipient matching, personalizing treatment plans, and improving immunosuppressive therapy dosing [1, 6].

International collaboration, fueled by globalization, has enhanced scientific cooperation across borders, facilitating the exchange of ideas and the integration of diverse knowledge to produce innovative outcomes [14, 15]. Several nations have strategically adopted international cooperation to enhance their domestic capacities and global competitiveness [14]. This collaborative strategy utilizes diverse viewpoints, specialized expertise, and access to broad patient populations, highlighting the significance of international partnerships in addressing distinct challenges [16].

Data sharing across borders is crucial for improving transplantation outcomes globally in the field of organ transplantation [16]. In the realm of utilizing AI within LKT, international collaboration is pivotal. It makes it possible to develop AI models that specifically address the various obstacles brought about by regional differences in genetics, racial demographics, environmental factors, and healthcare systems, in addition to meeting the need for transplants across the globe. These cooperative initiatives guarantee that AI solutions in LKT are inclusive, culturally aware, universally applicable, and precisely tailored to satisfy the complex requirements of transplant patients from different geographic locations.

Although AI applications in the field of LKT have undergone significant growth [6], to our knowledge, few efforts have quantified the degree of international collaboration through bibliographic analysis. This study addresses that gap by asking, “Are countries working together enough in the field of AI application in LKT?” Our analysis explores the extent of international collaboration and identifies emerging research trends.

Our findings emphasize the importance of international partnerships in progressing AI applications in LKT. We identified collaboration networks, significant institutions, and leading contributors. International co‐authorship accounted for 30.02% of publications, reflecting a substantial global effort to combine knowledge and resources, which is essential for the diverse and high‐quality advancement of AI in LKT. The United States of America was a prominent leader in publications and international partnerships, with strong intercountry connections. Collaborations are essential for advancing research, enabling access to diverse datasets and methodologies, and accelerating AI innovation and its incorporation into patient care globally.

The observed bibliometric patterns closely represent significant milestones in the growth of AI applications in the LKT field; the recent surge in publications starting in 2021 focusing on machine learning prediction models has demonstrated utility in predicting possible complications such as graft fibrosis, rejection, and long‐term survival. Moreover, from 2023, the advancement of research on AI‐driven prognostic models in liver transplantation coincides with initiatives to enhance patient selection criteria and waitlisting protocols, whereas in kidney transplantation, bibliometric analysis of relevant publications reflected the development of models to predict the course of immunosuppression regimens, organ allocation, and early graft dysfunction [17–19].

Integrating AI into LKT research, as in other medical fields, poses numerous challenges that highlight the complexities of encouraging successful international collaborations [6, 20]. Linguistic barriers and differing research priorities may still pose significant challenges, potentially limiting the extent and depth of global partnerships [15, 21]. Unequal funding compounds these issues, leading to unequal research settings with varying quality and breadth of datasets between different countries [20, 22]. In addition, the implementation of AI in healthcare, especially in LKT, faces technical and ethical challenges. Different countries′ varying data collection methods result in a mix of data formats and structures, posing challenges for the development of universally applicable and culturally sensitive AI models [20]. Furthermore, ethical concerns regarding data analysis and patient confidentiality increase the complexity of integrating AI into LKT [23]. Despite these barriers, the revolutionary impact of AI in this field is unquestionable. To overcome these challenges, a focused approach to collaboration and the creation of transparent, ethically sound AI instruments are required.

One notable limitation of this study is that the observed international collaboration, based on publication data, may not directly translate to the inclusion of diverse patient data in the research. The collaborations highlighted primarily reflect joint authorship and shared research efforts among countries, which do not necessarily indicate pooling or exchange of patient data. This gap points to a potential disconnect between international authorship collaboration and the actual pooling of diverse patient datasets. Despite this limitation, the observed degree of international collaboration presents a significant opportunity for enhancing transplant data sharing between countries. This sharing is crucial for capturing a wide range of patient demographics, including different ethnic groups and genetic backgrounds. By facilitating the exchange of transplant data across borders, researchers can work toward generalizing their findings, ensuring that AI applications in LKT are effective and applicable to diverse patient populations worldwide. Moreover, this study’s dependence on a single database may limit the generalizability of the results and potentially overlook relevant literature within the field of AI applications in LKT. In addition, the predominance of English‐language publications introduces the potential for language bias, as non‐English research may be underrepresented in the final dataset.

Future research should prioritize developing frameworks that enable wider sharing of patient data in international collaborations. This strategy will enhance the scope and effectiveness of AI in LKT and improve our comprehension of transplantation results for various populations. Creating AI tools that accurately represent the diverse global patient population can greatly promote inclusion and improve transplant success rates and the standard of patient care on a global scale. Furthermore, the growing emphasis on international collaboration stresses its significance for policy and practice, underscoring the necessity for policymakers and research institutions to actively endorse such partnerships. This support is essential for promoting the development of AI solutions that are effective and culturally sensitive, guaranteeing they cater to the requirements of diverse patient populations worldwide.

## 5. Conclusion

The integration of AI into LKT research is providing novel opportunities to enhance patient outcomes, decision‐making, and prediction. Nevertheless, the diversity of perspectives and patient data that are incorporated into existing models is restricted by the fact that current progress is primarily concentrated in high‐income countries. Although international collaboration is evident, the generalizability of current findings is restricted by the uneven distribution of participation. To ensure that future AI tools are inclusive, reliable, and applicable across diverse transplant populations, we must strengthen structured data sharing, engage underrepresented regions, and foster equitable, culturally adaptable frameworks.

## Author Contributions

Haneen Al‐Abdallat, MD: conceptualization, data analysis, manuscript writing, and revision. Noor Haj Mohammad, MD: data analysis, results interpretation, and manuscript writing. Ayham Asassfeh, MD: literature review, manuscript writing, and revision. Emily cooper, MD: manuscript writing and revision. Ayham Mohammad Hussein, MD: manuscript writing and revision. Mohammad Alzoubi. MD: supervision, conceptualization, and manuscript revision. Badi Rawashdeh, MD: supervision, conceptualization, and manuscript revision.

## Funding

No funding was received for this study.

## Conflicts of Interest

The authors declare no conflicts of interest.

## Data Availability

Data are available upon request.
